# Expression of *PEG11 *and *PEG11AS *transcripts in normal and callipyge sheep

**DOI:** 10.1186/1741-7007-2-17

**Published:** 2004-08-06

**Authors:** Christopher A Bidwell, Lauren N Kramer, Allison C Perkins, Tracy S Hadfield, Diane E Moody, Noelle E Cockett

**Affiliations:** 1 Department of Animal Sciences, Purdue University, 125 South Russell Street, West Lafayette, IN 47907-2042 USA; 2 Department of Animal, Dairy and Veterinary Sciences, College of Agriculture, Utah State University, 4800 Old Main Hill, Logan UT 84322-4800 USA

## Abstract

**Background:**

The callipyge mutation is located within an imprinted gene cluster on ovine chromosome 18. The callipyge trait exhibits polar overdominant inheritance due to the fact that only heterozygotes inheriting a mutant paternal allele (paternal heterozygotes) have a phenotype of muscle hypertrophy, reduced fat and a more compact skeleton. The mutation is a single A to G transition in an intergenic region that results in the increased expression of several genes within the imprinted cluster without changing their parent-of-origin allele-specific expression.

**Results:**

There was a significant effect of genotype (p < 0.0001) on the transcript abundance of *DLK1, PEG11*, and *MEG8 *in the muscles of lambs with the callipyge allele. *DLK1 *and *PEG11 *transcript levels were elevated in the hypertrophied muscles of paternal heterozygous animals relative to animals of the other three genotypes. The *PEG11 *locus produces a single 6.5 kb transcript and two smaller antisense strand transcripts, referred to as *PEG11AS*, in skeletal muscle. *PEG11AS *transcripts were detectable over a 5.5 kb region beginning 1.2 kb upstream of the *PEG11 *start codon and spanning the entire open reading frame. Analysis of *PEG11 *expression by quantitative PCR shows a 200-fold induction in the hypertrophied muscles of paternal heterozygous animals and a 13-fold induction in homozygous callipyge animals. *PEG11 *transcripts were 14-fold more abundant than *PEG11AS *transcripts in the gluteus medius of paternal heterozygous animals. *PEG11AS *transcripts were expressed at higher levels than *PEG11 *transcripts in the gluteus medius of animals of the other three genotypes.

**Conclusions:**

The effect of the callipyge mutation has been to alter the expression of *DLK1*, *GTL2*, *PEG11 *and *MEG8 *in the hypertrophied skeletal muscles. Transcript abundance of *DLK1 *and *PEG11 *was highest in paternal heterozygous animals and exhibited polar overdominant gene expression patterns; therefore, both genes are candidates for causing skeletal muscle hypertrophy. There was unique relationship of *PEG11 *and *PEG11AS *transcript abundance in the paternal heterozygous animals that suggests a RNA interference mechanism may have a role in *PEG11 *gene regulation and polar overdominance in callipyge sheep.

## Background

The mutation responsible for the callipyge trait is located within an imprinted gene cluster on the distal end of ovine chromosome 18 [1-3]. The callipyge phenotype is associated with an altered carcass composition including a 30–40% increase in muscle mass, a 6–7% decrease in carcass fat, decreased organ weights, and a more compact skeleton, all without a net affect on animal growth [4-7]. There is a pronounced hypertrophy of muscles in the loin and pelvic limbs and a lesser degree of hypertrophy in muscles of the thoracic limbs [6-8]. The callipyge phenotype is inherited in a non-Mendelian mode termed polar overdominance [9,10] in which only animals that inherit a normal allele (wild type; +) from the dam and the mutant callipyge allele from the sire (*CLPG*^*Pat*^) exhibit the callipyge phenotype. Maternal heterozygotes (*CLPG*^*Mat*^/+^*Pat*^) and callipyge allele homozygotes (*CLPG*^*Mat*^/*CLPG*^*Pat*^) have muscling and carcass compositions that are similar to normal sheep (wild type homozygotes; +/+).

**Figure 1:**
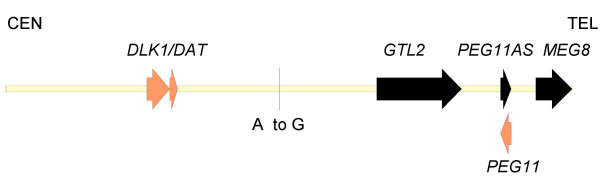
**The callipyge region of ovine chromosome 18. **A diagram of the callipyge region [3] based on GenBank accession No. AF345168 is shown. Six known transcripts are indicated along with the direction of transcription (arrows). Transcripts expressed from the paternal allele are shown as orange arrows and those expressed from the maternal allele are shown as black arrows [3]. A blue line indicates the position of the causative mutation. Alleles with an A are wild type (+) and the mutant callipyge allele (*CLPG*) has a G at this position [23, 24].

A physical contig spanning the region containing the callipyge mutation was constructed using overlapping ovine bacterial artificial chromosomes [11] and 215 kb of sequence was obtained from the contig [3]. Comparisons of the ovine sequence to the human genome sequence and to expressed sequence databases indicated the presence of at least six transcribed genes with allele-specific expression [3] (Figure 1). The gene order along the contig was found to be *Delta-like 1 *(*DLK1*), *DLK associated transcript *(*DAT*), g*ene-trap locus 2 *(*GTL2*), *paternal expressed gene 11 *(*PEG11 */ *PEG11AS*) and *maternal expressed gene 8 *(*MEG8*). The same conserved gene order was also found as an imprinted domain on human chromosome 14 and mouse chromosome 12 [12-15]. The *DLK1 *locus (also referred to as *PREF-1*, *Zog-1 *and *pG2*) encodes a transmembrane protein that contains epidermal-growth factor repeats [16-19]. Cleavage of the extracellular domain of *DLK1 *produces the circulating protein fetal antigen-1 [20]. *DAT *is a short non-coding RNA that has been proposed to be a cleavage product of extended *DLK1 *transcripts [21]. Both *DLK1*and *DAT *are expressed from the paternal allele [3,13-15]. *GTL2 *(also referred to as *MEG3*) and *MEG8 *genes express non-coding RNA from the maternal allele [3,13-15]. The *PEG11 *gene contains an intronless open reading frame of 1333 amino acids in sheep [3]. The human and mouse orthologues known as *retrotransposon-like 1 *(*RTL1*/*rtl1*) encode 1358 and 1745 amino acids, respectively. RNA transcripts were detected from the opposite strand of the same gene, referred to as *PEG11AS *(formerly known as *antiPEG11*) [3]. In the mouse, two maternally expressed microRNAs have been identified with perfect complementarity to mouse *Rtl1 *[22].

The causative mutation for callipyge is a single base transition of A (wild type; +) to G (*CLPG*) in the intergenic region located between *DLK1 *and *GTL2 *[23] (Figure 1). This mutation has been shown to be 100% concordant with all animals of the +^*Mat*^/*CLPG*^*Pat *^genotype based on haplotype analysis [23]. Analysis of sheep from 19 different breeds as well as 13 mammalian species revealed a highly conserved 12 base sequence that includes the single nucleotide polymorphism [24]. The G polymorphism is unique to direct descendents of the first known callipyge animal, a ram named "Solid Gold". This animal was mosaic for the mutation [24], providing strong evidence that this single nucleotide polymorphism is the causative mutation. Initial results indicate that the mutation alters the expression of several of the genes within the imprinted cluster [25,26] when they are inherited in *cis *without altering their parent-of-origin-specific expression [25].

In this study, we analyzed the expression of five genes within the callipyge cluster in the muscles of lambs of all four genotypes. Quantitative analysis of gene expression using a series of orthogonal contrasts showed that *DLK1*, *PEG11 *and *MEG8 *exhibited a polar overdominant pattern of gene expression. The expression of *PEG11 *and *PEG11AS *transcripts in the muscles of paternal heterozygous callipyge lambs (+^*Mat*^/*CLPG*^*Pat*^) was different from the other three genotypes. A sense/antisense interaction of *PEG11 *and *PEG11AS*, such as an RNA interference mechanism, would be consistent with a *trans *interaction between reciprocally imprinted genes that has been previously proposed as a mechanism for polar overdominance [27,28].

## Results

### Northern blot analysis

**Figure 2:**
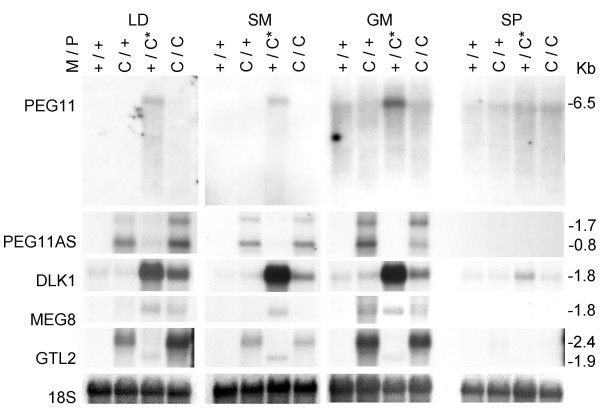
**Northern blot analysis of skeletal muscle RNA from 12-week-old lambs. **Expression of five genes from the callipyge region is shown for the four possible genotypes in 12-week-old lambs. Each lane contains total RNA from an individual animal. The genotypes are given with the maternal allele first followed by the paternal allele. The callipyge allele (*CLPG*) has been abbreviated to C and the paternal heterozygote that has the callipyge phenotype is indicated with an asterisk (*). Strand specific probes are indicated on the left and transcript sizes are indicated on the right. The skeletal muscles shown are the longissimus dorsi (LD), semimembranosus (SM), gluteus medius (GM) and the supraspinatus (SP). The blots were hybridized with an 18S RNA probe to show the equivalence of RNA loading and transfer.

Muscle samples were collected from 12- and 8-week-old lambs, when muscle hypertrophy is well established in animals with the callipyge phenotype. Total RNA was extracted from three muscles that undergo hypertrophy including longissimus dorsi (loin), semimembranosus, and gluteus medius (pelvic limb), and one muscle that does not undergo hypertrophy, the supraspinatus, (thoracic limb). Strand specific probes were used to analyze *PEG11 *and *PEG11AS *expression. Hybridization of longissimus dorsi, semimembranosus and gluteus medius northern blots with a *PEG11 *probe indicated expression of a 6.5 kb *PEG11 *transcript in paternal heterozygotes (+^*Mat*^/*CLPG*^*Pat*^) that was not readily detectable in the other three genotypes (Figure 2). Two smaller *PEG11AS *transcripts of 1.7 kb and 0.8 kb were detected in the two genotypes with maternally inherited callipyge alleles (*CLPG*^*Mat*^/+^*Pat *^and *CLPG*^*Mat*^/*CLPG*^*Pat*^) by a probe from the complementary strand. *PEG11 *and *PEG11AS *transcripts were not detected in the supraspinatus. The expression of *DLK1 *was detectable at various levels in each of the four muscles and all four genotypes, although *DLK1 *appeared to be up-regulated in the loin and pelvic limb muscles of +^*Mat*^/*CLPG*^*Pat *^and *CLPG*^*Mat*^/*CLPG*^*Pat *^animals (Figure 2). *GTL2 *transcripts of around 2.4 kb were evident in the *CLPG*^*Mat*^/+^*Pat *^and *CLPG*^*Mat*^/*CLPG*^*Pat *^genotypes. A distinct 1.9 kb *GTL2 *transcript was consistently detected in the +^*Mat*^/*CLPG*^*Pat *^genotype and no *GTL2 *transcripts were detected in the +/+ genotype. A 1.8 kb *MEG8 *transcript was expressed to a lesser degree than *GTL2 *in the *CLPG*^*Mat*^/+^*Pat *^and *CLPG*^*Mat*^/*CLPG*^*Pat *^genotypes and was also detectable in the +^*Mat*^/*CLPG*^*Pat *^genotype. The expression pattern of *MEG8 *was the least consistent in the northern blot analysis of individuals of the four genotypes.

### *PEG11/PEG11AS*

**Figure 3:**
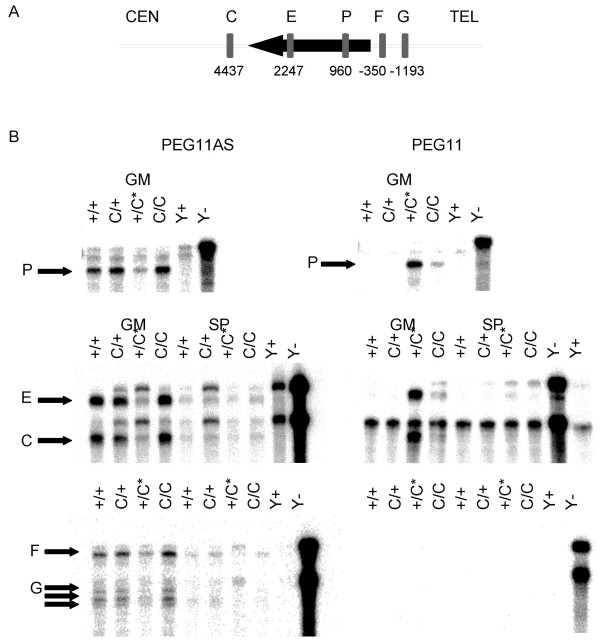
**Mapping of *PEG11 *and *PEG11AS *transcripts by ribonuclease protection assay. **A) A diagram of the *PEG11 *open reading frame (black arrow) and the position of the five riboprobes are shown. The *PEG11 *open reading frame extends from base 191859 to 187858 on the complementary strand of AF354168. B) Ribonuclease protection assays using *PEG11AS *(left column) and *PEG11 *(right column) are shown using total RNA from the gluteus medius (GM) and the supraspinatus (SP) muscles for each of the four possible genotypes. The genotypes are given with the maternal allele first followed by the paternal allele. The *CLPG *allele has been abbreviated to C and the paternal heterozygote that demonstrates the callipyge phenotype is indicated with an asterisk (*). Yeast RNA with RNase treatment (Y+) and without RNase treatment (Y-) are shown as controls. A black arrow indicates the RNA fragments protected by each of the five RNA probes. Some full-length probes for C and E that could not be completely eliminated by RNase digestion are present in both muscle RNA samples and the yeast RNA control (Y+).

Ribonuclease protection assays were performed using five riboprobes to map the *PEG11 *and *PEG11AS *transcripts to the contig sequence (Figure 3A). The results for riboprobe P confirm the expression of *PEG11 *transcripts in the gluteus medius of +^*Mat*^/*CLPG*^*Pat *^animals and showed a very low level of *PEG11 *in the gluteus medius of *CLPG*^*Mat*^*/CLPG*^*Pat *^animals. *PEG11AS *transcripts were readily detectable in animals of the +/+, *CLPG*^*Mat*^/+^*Pat *^and *CLPG*^*Mat*^/*CLPG*^*Pat *^genotypes but were slightly above background levels in the +^*Mat*^/*CLPG*^*Pat *^animals (Figure 3B). Two other *PEG11 *probes from the open reading frame (E) and from the 3'UTR (C) showed equivalent results to riboprobe P in the gluteus medius (Figure 3B). The same expression pattern was seen for *PEG11 *and *PEG11AS *transcripts in the supraspinatus, but signal from the protected riboprobes was just above background level.

Two probes from upstream of the *PEG11 *coding sequence (riboprobes F and G) did not detect any *PEG11 *transcripts, indicating that transcription of the 6.5 kb *PEG11 *was initiated within 350 bp of the start codon of the open reading frame. Riboprobe F detected *PEG11AS *transcripts in the gluteus medius and supraspinatus of all four genotypes, with the lowest expression in the paternal heterozygous (+^*Mat*^/*CLPG*^*Pat*^) animals. The *PEG11AS *riboprobe G shows three protected fragments, suggesting variable splice junctions or transcription termination sites for *PEG11AS *transcripts.

### Quantitative analysis of the effect of genotype on gene expression

**Figure 4:**
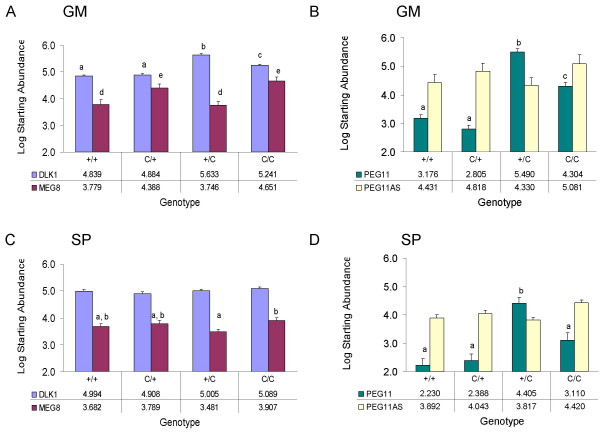
**Quantitative reverse transcriptase PCR analysis of transcript abundance in the gluteus medius and supraspinatus muscles. **Least square means and standard errors for transcript abundance by genotype are shown for the gluteus medius (GM), and supraspinatus (SP). The genotypes are given with the maternal allele first followed by the paternal allele and the *CLPG *allele has been abbreviated to C. Quantification of *DLK1 *and *MEG8 *transcripts (A, C) was based on random primed cDNA synthesis using 213 ng of total RNA. Quantification of *PEG11 *and *PEG11AS *(B, D) transcripts was based on gene-specific priming of cDNA synthesis using 400 ng of total RNA. Different superscripts indicate significant differences (p < 0.05) between genotypic means for a given mRNA transcript and muscle. Numerical values for the log starting abundance are given for each mRNA transcript in a table below the genotypes.

Gene expression was measured in the gluteus medius and supraspinatus of 8 week-old animals (Figure 4) using quantitative PCR. The expression of glyceraldehyde-3-phosphate dehydrogenase was not significantly different across the four genotypes in the gluteus medius and supraspinatus (Table 1), indicating that equivalent amounts of RNA were used for cDNA synthesis and quantitative PCR. The effect of the callipyge mutation on genotype-specific expression of *DLK1 *and *PEG11 *in gluteus medius was the same (Table 1) although the magnitude of the response was greater for *PEG11 *than *DLK1 *(Figure 4A and 4B). The paternal heterozygous (+^*Mat*^/*CLPG*^*Pat*^) animals had the highest transcript abundance (p < 0.05), followed by *CLPG*^*Mat*^/*CLPG*^*Pat *^animals, which had significantly greater transcript abundance (p < 0.05) than *CLPG*^*Mat*^/+^*Pat *^or +/+ animals. The mRNA abundance of *DLK1 *in the gluteus medius was 6-fold and 2.5-fold greater in +^*Mat*^/*CLPG*^*Pat *^lambs and *CLPG*^*Mat*^*/CLPG*^*Pat *^lambs respectively, relative to normal lambs (+/+; Figure 4A). *PEG11 *mRNA abundance in the gluteus medius was 200-fold and 13-fold greater in +^*Mat*^/*CLPG*^*Pat *^lambs and *CLPG*^*Mat*^/*CLPG*^*Pat *^lambs respectively, relative to normal lambs (+/+; Figure 4B).

The effect of the callipyge mutation on *DLK1 *and *PEG11 *expression in the supraspinatus was different (Table 1). No differences in *DLK1 *transcript abundance in the supraspinatus were found among the four genotypes (Figure 4C). Although not statistically analyzed, the level of *DLK1 *expression was similar between the gluteus medius and supraspinatus (Figure 4A and 4C). *PEG11 *expression in the supraspinatus had a different genotype-specific pattern than in the gluteus medius. PEG11 expression was elevated 150-fold in +^*Mat*^/*CLPG*^*Pat *^animals (p < 0.05) but was not significantly changed in the other three genotypes (Figure 4D). The transcript abundance of *PEG11 *in supraspinatus was generally much lower than in gluteus medius of the same genotype. *PEG11 *expression in the supraspinatus was 12-fold lower than in the gluteus medius of paternal heterozygous animals (Figure 4B and 4D).

Maternal inheritance of the callipyge allele significantly altered the expression of *MEG8 *but not *PEG11AS *in the gluteus medius (Table 1). Transcript abundance of *MEG8 *in gluteus medius was 6-fold greater (p < 0.05) in the *CLPG*^*Mat*^/+^*Pat *^and *CLPG*^*Mat*^/*CLPG*^*Pat *^animals relative to the +^*Mat*^/*CLPG*^*Pat *^and +/+ animals (Figure 4A). Expression of *MEG8 *in the supraspinatus was also affected by the callipyge mutation but to a lesser degree (Figure 4C). The homozygous callipyge animals had significantly higher *MEG8 *transcript abundance than paternal heterozygotes, but those two genotypes were not significantly different from maternal heterozygotes and homozygous wild type lambs.

Orthogonal contrasts were used to analyze different models of gene action for the genes that had a significant effect for genotype (Table 1). The polar overdominance contrast was significant for transcript abundances of *DLK1*, *PEG11 *and *MEG8 *in gluteus medius and for *PEG11 *and *MEG8 *in the supraspinatus. The additive contrasts were also significant for *DLK1*, *PEG11 *and *MEG8 *in gluteus medius but were only significant for *PEG11 *in supraspinatus. The maternal dominance contrast was significant for *DLK1 *and *PEG11 *gluteus medius.

## Discussion

Our results show a clear pattern of increased expression for genes within the imprinted callipyge cluster in muscles that become hypertrophied, whereas expression of these genes was either reduced or absent in a muscle that does not become hypertrophied. The increased gene expression occurred when the mutation was inherited in *cis *and was dependent on each gene's imprinting status, consistent with previous reports [25,26]. These results support the hypothesis that the mutation has disrupted a long range control element [27]. Two paternally expressed genes, *DLK1 *and *PEG11*, had significantly increased transcript abundance when a callipyge allele was inherited from the sire. One maternally expressed gene, *MEG8*, showed significantly increased transcript abundance when the callipyge allele was inherited from the dam. The changes in gene expression were sustained until 12 weeks of age, when the differences in growth and body composition between callipyge and normal lamb are established and are subsequently maintained [7].

Northern blot analysis suggests that expression of *GTL2 *and *PEG11AS *was increased by maternal inheritance of the callipyge allele. Quantitative analysis was not done for *GTL2 *in this study due to the expression of numerous alternatively spliced transcripts that have been reported for mice and sheep [26,29]. The 2.4 kb *GTL2 *mRNA seen in this study consisted of a heterogeneous population of alternatively spliced mRNAs. Similarly, two *PEG11AS *transcripts were detected over a 5.6 kb area extending from beyond the 5' end of the *PEG11 *transcript to the 3'UTR. The 1.7 and 0.8 kb *PEG11AS *transcripts detected by northern blot analysis using probe P would not be protected by probes C or G unless the *PEG11AS *transcripts undergo intron splicing or there are other transcripts that were not detected in the northern blots. Therefore, the effect of the callipyge mutation on *GTL2 *and *PEG11AS *will require a more extensive analysis to fully elucidate their expression patterns in the four genotypes and determine their role in the callipyge model.

Due to their paternal allele-specific expression, the *DLK1 *and *PEG11 *genes are both candidates for an effector gene that is responsible for the skeletal muscle hypertrophy exhibited by paternal heterozygous (+^*Mat*^/*CLPG*^*Pat*^) animals. In this study, both genes showed a polar overdominant expression pattern in that paternal heterozygotes had significantly higher levels of gene expression than the other three genotypes. The major differences between *DLK1 *and *PEG11 *were the magnitude and the muscle specificity of the up-regulation. *DLK1 *was readily detectable in all muscles and genotypes by northern blot, but the up-regulation in paternal heterozygous animals was restricted to muscles of the loin and pelvic limb. The quantitative results showed a 6-fold increase in *DLK1 *transcript abundance in the gluteus medius and no change of *DLK1 *abundance in the supraspinatus. This pattern of gene expression is consistent with studies on individual muscle growth that show significant muscle hypertrophy in the gluteus medius but not in the supraspinatus [6,8]. *DLK1 *transcripts were significantly increased (2.5-fold) in the gluteus medius of *CLPG*^*Mat*^/*CLPG*^*Pat *^animals, which do not exhibit muscle hypertrophy. The lack of a phenotype in *CLPG*^*Mat*^/*CLPG*^*Pat *^animals could be due to a threshold effect that requires more than a 2.5-fold increase in *DLK1 *transcript abundance to change muscle growth.

The expression of *PEG11 *was very low in the gluteus medius and supraspinatus of normal sheep and was induced in both muscles of callipyge lambs. Northern blot analysis, ribonuclease protection assay and quantitative PCR results all show that the expression of *PEG11 *and *PEG11AS *transcripts was much lower in the supraspinatus than the other muscles. The high level of expression of *PEG11 *in the gluteus medius relative to the supraspinatus of callipyge lambs indicates that *PEG11 *could also be the gene responsible for muscle hypertrophy if there is a threshold level required to change muscle growth. The *PEG11 *gene has a long intronless open reading frame but it is not known if a protein is produced or what function it may have.

The overdominant nature of the callipyge phenotype through the lack of muscle hypertrophy in animals with the *CLPG*^*Mat*^/*CLPG*^*Pat *^genotype is one of the more intriguing aspects of the trait and has led to a hypothesis of *trans *effects by other reciprocally imprinted genes in the callipyge region [25,27]. If the *PEG11 *gene has a direct role in muscle hypertrophy, either alone or in concert with *DLK1*, then *PEG11AS *may have a role in a *trans *effect on *PEG11 *expression. In +^*Mat*^/*CLPG*^*Pat *^animals, *PEG11 *transcripts were 14-fold and 4-fold more abundant than *PEG11AS *transcripts in the gluteus medius and supraspinatus respectively. *PEG11AS *transcripts were more abundant than *PEG11 *transcripts in the other three genotypes for both muscles. Therefore, the relative abundance of *PEG11 *transcripts to *PEG11AS *transcripts was unique in paternal heterozygous animals. MicroRNAs are a central component of RNA interference mechanisms. In the mouse, two antisense microRNA have been identified for the orthologous *rtl1 *locus [22]. RNA interference mechanisms have been shown both to repress transcription by inducing heterochromatin formation [30-32] and to cause post-transcriptional silencing through nuclear retention or targeted degradation [33-35]. MicroRNAs may be produced from post-transcriptional processing of *PEG11AS *RNA and be involved in normal regulation of the locus and in generating overdominance [28]. Expression of *PEG11AS *may normally cause repression of the paternal *PEG11 *locus since very little *PEG11 *mRNA was detectable in the muscles of normal animals. In the animals with a paternally inherited callipyge allele, (+^*Mat*^/*CLPG*^*Pat *^and *CLPG*^*Mat*^/*CLPG*^*Pat*^), the normal repression of the *PEG11 *locus has been disrupted by the mutation in a putative long range control element [27]. *PEG11 *transcripts only accumulate in significant excess of *PEG11AS *in the paternal heterozygous animals, whereas in the *CLPG*^*Mat*^/*CLPG*^*Pat *^animals the *PEG11AS *transcripts remain in excess and may prevent the accumulation of the threshold level of *PEG11 *mRNA required to produce a muscle hypertrophy phenotype. Although the expression of *PEG11AS *was not affected by genotype in quantitative PCR, the northern blots and transcript mapping indicate there are multiple transcripts that are likely to undergo different intron splicing. Further analysis of *PEG11AS *expression will be necessary to determine its role in the *PEG11 *locus regulation.

## Conclusions

The effect of the callipyge mutation has been to increase transcript abundance of four genes, *DLK1*, *GTL2*, *PEG11 *and *MEG8*, within the imprinted cluster in skeletal muscles that become hypertrophied. The increase in transcript abundance was consistent with each gene's parental allele-specific expression. The *DLK1 *and *PEG11 *genes were both expressed at their highest levels in paternal heterozygous animals and exhibited polar overdominant gene expression patterns. Therefore, both genes are candidates for causing muscle hypertrophy. *DLK1 *expression was only elevated in muscles that undergo hypertrophy, so its muscle-specific increase was consistent with the callipyge phenotype. *PEG11 *was 12-fold more abundant in hypertrophied muscle than non-hypertrophied muscle and only paternal heterozygous animals had *PEG11 *transcript levels in excess of *PEG11AS *transcript levels. The unique relationship of *PEG11 *and *PEG11AS *in paternal heterozygous animals suggests that an RNA interference mechanism may have a role in regulating the *PEG11 *locus and polar overdominance in callipyge sheep.

## Methods

### Sample collection

A series of planned matings were conducted to produce the four possible callipyge genotypes. The genotypes of all lambs were verified using the single nucleotide polymorphism [23,24] and several markers that flank the callipyge region on chromosome 18. Lambs were slaughtered in accordance with humane practices approved by the Utah State University Institutional Animal Care and Use Committee. Samples were collected from the longissimus dorsi, semimembranosus, gluteus medius and supraspinatus, and preserved in RNAlater (Ambion Inc., Woodlands, TX USA). The tissue samples were homogenized in 4 M guanidinium thiocyanate, 25 mM sodium citrate, 50 mM EDTA, 1% sodium-N-lauroyl-sarcosine and total RNA was sedimented by ultracentrifugation of the homogenate on a cushion of 5.7 M CsCl, 50 mM EDTA [36]. Purified RNA was quantified by spectrophotometry and the use of a constant mass of RNA for each quantitative assay was based on absorbance at 260 nm. Total RNA was treated with DNase I using DNA *free*™ reagents (Ambion Inc.) to remove trace genomic DNA prior to use in the ribonuclease protection assay and quantitative PCR.

### Northern blot analysis

Northern blots were prepared using denaturing formaldehyde gel electrophoresis (NorthernMax™; Ambion Inc) of 10 μg of total RNA and transferred to positively charged nylon membranes using standard methods [37]. Primer sequences used to amplify probes from the callipyge region are given in Charlier *et al*. [25], and the PCR products were verified by DNA sequencing. Strand specific DNA probes were synthesized by 40 cycles of asymmetric PCR with Strip-EZ™ nucleotides (Ambion Inc.) and 50 μCi of α-[^32^P]-dATP (Amersham-Pharmacia, Piscataway, NJ USA). Unincorporated nucleotides were removed by spin column chromatography (BioSpin P30; Bio-Rad Inc., Hercules, CA USA). The probes were hybridized to the membranes without denaturation using Ultrahyb™ (Ambion Inc.) at 42°C overnight. After hybridization, the membranes were washed in 2X SSC (0.3 M sodium chloride, 0.03 M sodium citrate)/0.5% SDS followed by 3 washes in 1X SSC/ 0.1% SDS at 65°C for 30 min and a final high stringency wash in 0.1X SSC/ 0.1%SDS at 65°C for 30 min. The northern blots were exposed to Kodak XAR autoradiography film for 18 to 72 h at -80°C. After autoradiography, the probe was degraded and removed from the membranes using Strip-EZ™ reagents (Ambion Inc.).

### Ribonuclease protection assay

Templates for synthesizing strand specific RNA probes for the ribonuclease protection assay (RPA) were generated from PCR products (Table 2) by ligation of double stranded oligonucleotides containing T7 or SP6 promoter sequences and re-amplification with an adapter and gene specific primer (Lig'n Scribe™, Ambion Inc.). Labeled RNA probes were synthesized using MAXIscript™ reagents (Ambion, Inc.) and 50 μCi of α-[^32^P]-UTP (Amersham-Pharmacia). The RPA were conducted using RPA III™ reagents (Ambion Inc.) and standard urea/acrylamide gel electrophoresis methods [37]. Dried gels were exposed to phosphorimaging screens and images were collected using a Cyclone Storage Phosphorimager (Packard Instrument Co., Meriden, CT USA).

### Quantitative PCR

Complementary DNA was synthesized from 3.2 μg of total RNA using random hexamer priming and MMLV reverse transcriptase reagents (Invitrogen, Carlsbad, CA USA) with RNase inhibitor supplementation (Superase Inhibitor, Ambion Inc). The cDNA samples were diluted with water and aliquoted so that the quantification was based on 213 ng of total RNA for analysis of glyceraldehyde-3-phosphate dehydrogenase, DLK1 and MEG8 transcripts. PEG11 and PEG11AS transcripts were measured using gene-specific priming of cDNA synthesis from 1.6 μg of total RNA. The cDNA was diluted with water and aliquoted for quantification based on 400 ng of total RNA. Each cDNA sample was amplified in triplicate using the SYBR Green Jump Start™ system (Sigma-Aldridge, St. Louis, MO USA). Quantification standards were composed of aliquots of plasmids containing target PCR products in 10-fold serial dilutions ranging from 10^8 ^to 10^2 ^molecules. The standards were used to calculate a regression of threshold cycle on molecule copy number to determine a log value of starting abundance for each of the cDNA samples based on their threshold cycle. The PCR reactions were run for 40 cycles in an iCycler Real-Time PCR Detection System (Bio-Rad Inc.). The log value of starting abundance for each gene was analyzed by analysis of variance using the PROC MIXED procedure of SAS [38]. The analysis model included genotype as a fixed effect and animal within genotype as a random effect. The number of animals representing each genotype (6 to 8) is given in Table 3. Orthogonal contrasts were used to evaluate different models of gene action if the effect of genotype on log value of starting abundance was significant. Initially, traditional additive, dominance and reciprocal heterozygote effects were evaluated (see Table 3 for contrasts). If the reciprocal heterozygote effect was significant (p < 0.05), a second set of orthogonal contrasts was used to test additive, maternal dominance and polar overdominance effects as previously described by Freking *et al*. [10] (Table 3).

## Authors' contributions

CB participated in planning the study and developing the experimental design, conducted the northern blot analysis and ribonuclease protection assays, and wrote the drafts of the manuscript. LK and AP isolated RNA and performed the quantitative PCR assays. TH collected the muscle samples and genotyped the animals used in this study. DM participated in experimental design and performed the statistical analysis. NC participated in planning the study, set up a series of matings to generate all four genotypes and provided the experimental animals. All authors read and approved the final manuscript.

## Figures and Tables

**Table 1:** Statistical analysis of gene expression by quantitative PCR

			Orthogonal Contrast P-Values		
			
Muscle	Gene	Effect of Genotype (p-values)	Additive	Maternal Dominance	Paternal Polar Overdominance
GM	*DLK1*	0.0001	0.0001	0.0261	0.0001
	*MEG8*	0.0005	0.0009	0.4083	0.0047
	*PEG11*	0.0001	0.0001	0.0001	0.0001
	*PEG11AS*	0.2622			
	*G3PD*	0.1393			
SP	*DLK1*	0.1865			
	*MEG8*	0.0325	0.1398	0.9665	0.0094
	*PEG11*	0.0001	0.0179	0.3315	0.0001
	*PEG11AS*	0.1438			
	*G3PD*	0.3626			

**Table 2:** Primer sequences for ribonuclease protection assay probes and quantitative PCR

			Size^a^	T_m_	Location^b^
Primer		Sequence	bp	°C	
PEG11/PEG11AS					
C	F	AGGAACACCGCTGTGGAGGTAGAA	135	59	4337
	R	ACAGCAGAGGCAGCCAAGCA			
E	F	GGTGACGCCCGTCTGCAAGT	180	61	2427
	R	GTGGAACGGTTCGCCGACAT			
P^c^	F	ACAGCTCAACAGTGGAGGTCATG	199	55	996
	R	ATCAGCTGGCAGAGCACGATGAAC			
F	F	TTCCCCCATGGCTGTGAGAAAT	175	58	-390
	R	CCTCTGTGACCTTCTGGTGACCAA			
G	F	AGGCTGAATTGACAGAGATGT	124	49	-1193
	R	GTTAAATGGCTCAAGAACGA			
*DLK1*	F	CCCGTCCTCTTGCTCCTGCT	116	58	
	R	GGCTGGCACCTGCACACACT			
*MEG8*	F	CCCAGGGAGTGTGAGGCTCTTCT	100	56	
	R	GGACCCACGGCTGACCTGTT			
*G3PD*	F	TGAGTGTCGCTGTTGAAGT	150	58	
	R	CCTGCCAAGTATGATGAGAT			

^a^Size of the PCR product and protected fragment for RPA ^b^Location for the PCR product is relative to the first base of the *PEG11 *open reading frame. ^c^Charlier *et al*. [3]

**Table 3:** Statistical methods for genetic models

	Orthogonal Contrast Values by Genotype			
Genetic Model	+/+	*CLPG*^*Mat*^/+^*Pat*^	+^*Mat*^/*CLPG*^*Pat*^	*CLPG*^*Mat*^/*CLPG*^*Pat*^

Additive	-1	0	0	1
Dominance	-1	1	1	-1
Reciprocal Heterozygote	0	-1	1	0
Maternal Dominance	-1	2	0	-1
Paternal Polar Overdominance	-1	-1	3	-1
Number of Animals	6	7	8	8
